# Long-Term Sleep Data Among Older Adults: Atherosclerosis Risk in Communities Neurocognitive Study

**DOI:** 10.1093/geroni/igaf122.1963

**Published:** 2025-12-31

**Authors:** Erin Spaulding, Henry Zhao, Jie Ding, Pamela Lutsey, Brendan Lucey, Katherine Ornstein, Josef Coresh, Seth Martin

**Affiliations:** Johns Hopkins University, Baltimore, Maryland, United States; Johns Hopkins University School of Medicine, Baltimore, Maryland, United States; Johns Hopkins University School of Medicine, Baltimore, Maryland, United States; University of Minnesota, Minneapolis, Minnesota, United States; WashU Medicine Department of Neurology, St. Louis, Missouri, United States; Johns Hopkins University, Baltimore, Maryland, United States; New York University, New York, New York, United States; Johns Hopkins University School of Medicine, Baltimore, Maryland, United States

## Abstract

The American Heart Association recommends 7-9 hours of sleep per night, based primarily on self-reported data, to promote cardiovascular and cognitive health. Due to limited objective, long-term sleep data in adults aged 80 years and older, we sought to objectively characterize sleep in this group. Fitbit Charge 6 devices were used to collect long-term data on sleep among Atherosclerosis Risk in Communities Neurocognitive Study (ARIC NCS) participants with a smartphone in 2024-2025. Nighttime sleep (8 pm-8 am) with ≥8 hours of heart rate data, ensuring consistent Fitbit wear, was analyzed to assess sleep duration and efficiency. Multiple linear regression was used to assess independent predictors of sleep efficiency, including age, sex, and race. A total of 301 ARIC NCS participants (84.0±3.2 years [36% ≥85 years], 60% women, 82% White) contributed a median of 112 (IQR: 144) nights of sleep data (∼3.7 months). This sample of older adults aged 79-95 years spent a median of 301 (IQR: 115) minutes asleep (∼5 hours) and 450 (IQR: 104) minutes in bed (∼7.5 hours) per night. Median sleep efficiency was 68% (IQR: 15), with 85% having fair or poor sleep (efficiency <80%). Men (-4.28%; 95% CI: -7.11%, -1.45%) and Black adults (-5.40%; 95% CI: -9.01%, -1.80%) had significantly lower sleep efficiency compared to women and White adults. In conclusion, adults 80 years and older, particularly Black adults and men, experience poor sleep. Future research could examine long-term sleep variability in this population and reasons for sleep deficits among Black older adults and men.

